# *Salmonella enterica* subsp*. enterica* Welikade: guideline for phylogenetic analysis of serovars rarely involved in foodborne outbreaks

**DOI:** 10.1186/s12864-022-08439-2

**Published:** 2022-03-19

**Authors:** Emeline Cherchame, Laurent Guillier, Renaud Lailler, Marie-Leone Vignaud, Nathalie Jourdan-Da Silva, Simon Le Hello, François-Xavier Weill, Sabrina Cadel-Six

**Affiliations:** 1 Laboratory for Food Safety, French Agency for Food, Environmental and Occupational Health & Safety (ANSES), 94700 Maisons-Alfort, France; 2 Present address: Data Analysis Core, Paris Brain Institute, ICM, Paris, France; 3 Santé Publique France, 94410 Saint-Maurice, France; 4 Centre National de Référence Des Escherichia Coli, Institut Pasteur, Unité Des Bactéries Pathogènes Entériques, Shigella et Salmonella, 75015 Paris, France; 5 Present address: Groupe de Recherche Sur L’Adaptation Microbienne (GRAM 2.0), Normandie Univ, UNICAEN, Caen, France

**Keywords:** *Salmonella* Welikade, Reference genome, Outbreak characterization, EnteroBase analysis, Core genome SNP analysis, ClonalFrameML, Accessory genome analysis, Phage analysis, Virulome analysis

## Abstract

**Background:**

*Salmonella* spp. is a major foodborne pathogen with a wide variety of serovars associated with human cases and food sources. Nevertheless, in Europe a panel of ten serovars is responsible for up to 80% of confirmed human cases. Clustering studies by single nucleotide polymorphism (SNP) core-genome phylogenetic analysis of outbreaks due to these major serovars are simplified by the availability of many complete genomes in the free access databases. This is not the case for outbreaks due to less common serovars, such as Welikade, for which no reference genomes are available. In this study, we propose a method to solve this problem. We propose to perform a core genome MLST (cgMLST) analysis based on hierarchical clustering using the free-access EnteroBase to select the most suitable genome to use as a reference for SNP phylogenetic analysis. In this study, we applied this protocol to a retrospective analysis of a *Salmonella enterica* serovar Welikade (*S*. Welikade) foodborne outbreak that occurred in France in 2016. Finally, we compared the cgMLST and SNP analyses. SNP phylogenetic reconstruction was carried out considering the effect of recombination events identified by the ClonalFrameML tool. The accessory genome was also explored by phage content and virulome analyses.

**Results:**

Our findings revealed high clustering concordance using cgMLST and SNP analyses. Nevertheless, SNP analysis allowed for better assessment of the genetic distance among strains. The results revealed epidemic clones of *S*. Welikade circulating within the poultry and dairy sectors in France, responsible for sporadic and non-sporadic human cases between 2012 and 2019.

**Conclusions:**

This study increases knowledge on this poorly described serovar and enriches public genome databases with 42 genomes from human and non-human *S*. Welikade strains, including the isolate collected in 1956 in Sri Lanka, which gave the name to this serovar. This is the first genomic analysis of an outbreak due to S. Welikade described to date.

**Supplementary Information:**

The online version contains supplementary material available at 10.1186/s12864-022-08439-2.

## Background

*Salmonella* spp. is a foodborne bacterium responsible for several hundred million human cases each year worldwide. In Europe, between 2015 and 2019, an average of 92,119 confirmed human cases were reported annually by the European Centre for Disease Prevention and Control (ECDC). Importantly, 10 major serovars (*Salmonella enterica* serovars Agona, Enteritidis, Infantis, Kentucky, Derby, Newport, Stanley, Typhimurium, 1,4,[5],12:i:- and Virchow) were responsible for 80% of these human infections [1–5]. In France, these 10 serovars were responsible for around 90% of human salmonellosis, with *Salmonella enterica* serovars Enteritidis, Typhimurium and 1,4,[5],12:i:- alone accounting for 71% of infections [6–9]. Among the remaining 10% of human salmonellosis cases not related to these 10 major serovars in France, serovars such as *S. enterica* serovar Welikade (hereafter referred to as *S.* Welikade) can be responsible for sporadic cases or outbreaks. *S.* Welikade has been poorly described worldwide, with a few publications citing strains isolated in Sri Lanka [10], Australia [11] or Sweden [12]. It is also not included in the annual reports of the European Food Safety Authority (EFSA) [1–5], and is underrepresented in open-access genome databases, such as NCBI [13] and EnteroBase [14] (i.e. 11 genomes available as of December 2020).

In France, during summer 2016, an *S*. Welikade outbreak was detected by the French Public Health Agency (*Santé publique France*; SPF) and the French National Reference Center for *Escherichia coli*, *Shigella* and *Salmonella* (NRC). The NRC recorded eight human cases likely associated with this outbreak. For the period between 2012 and 2019, the NRC database contains 5 other sporadic *S.* Welikade isolates from human cases. The epidemiological investigation carried out by the Regional Office of SPF in the Occitanie region, where five of these cases occurred, identified the consumption of goat’s cheese made with raw milk as the source of the outbreak. During summer 2016, the non-human *Salmonella* Network (SN) [15], coordinated by the French Laboratory of Food Safety, received one *S*. Welikade strain from goat’s cheese. SN data for the period between 2012 and 2019 showed that the prevalence of *S.* Welikade among non-human SN strains was 0.04%, with only 110 isolates mainly collected in the Auvergne-Rhône-Alpes region (neighboring Occitanie to the north) from the poultry sector, especially from laying hens and broilers (*Gallus gallus*) [16]. To better document the population structure and transmission chains of *S.* Welikade in France, we undertook a genomic epidemiology analysis of all the human strains isolated since 2012 and a spatiotemporal representative panel of non-human strains isolated during the same period.

SNP phylogenetic core-genome analysis is a well-known method that makes it possible to cluster strains suspected of being related and to quantify the genomic distance between genomes in outbreak investigations and in studies of sporadic infections [17–19]. Phylogenetic SNP analysis requires a complete reference genome for the mapping analysis step and to calculate the pairwise distance between genomes. Nevertheless, in the context of investigations related to rare serovars, it may be difficult to identify a reference genome to use for the analysis.

In this study, we propose to solve this problem by analyzing *S*. Welikade strains by core-genome multilocus sequence type (cgMLST) phylogenetic analysis in the free-access EnteroBase [14, 20]. The cgMLST analysis allowed us to identify the closest and most suitable complete genome to use for SNP phylogenetic analysis and to record other *S.* Welikade strains isolated all over the world. Finally, we carried out a SNP core genome phylogenic analysis on 52 *S*. Welikade genomes, 41 isolated in France over 7 years, including all strains isolated from human infections. We also undertook accessory genome analyses to identify genomic factors characterizing the epidemic clones identified. This study enabled high-resolution molecular typing of 52 *S*. Welikade genomes and enriched the free-access genome databases.

## Results

### EnteroBase Hierarchical clustering and cgMLST analyses

Among the 42 human and non-human *S*. Welikade strains analyzed, 14 were from the NRC and 28 from the SN. The 14 strains originating from the NRC included (i) the historical *S*. Welikade strain, isolated for the first time in Sri Lanka in 1956 [21], (ii) 12 strains corresponding to all the clinical *S*. Welikade cases that occurred in metropolitan France since 2012, and (iii) one strain isolated from goat’s cheese in 2016. Among the panel of 28 non-human strains, all non-human strains isolated in 2016, the year of the *S*. Welikade outbreak, were included (*n* = 8). The other twenty non-human strains were chosen, among the 110 collected between 2012 and 2019, with particular attention to the geographic distribution and the sector of sample collection (i.e. the proportion of *S*. Welikade in the animal, food and feed sectors within the SN collection database was used to make the selection) (Supplementary Fig. 1).

The dataset of 42 *S*. Welikade strains from France, raw reads, and metadata were uploaded to EnteroBase [22] and various markers were studied. Among these 42 strains, 41 had MLST [23] profile ST3300 and one ST6416 (i.e. the 839 K historical strain isolated in Sri Lanka). In the EnteroBase *Salmonella* database [14, 20], 11 other *S*. Welikade genomes (based on predicted serotype by SISTR [24]) were available from five continents: Europe (*n* = 3), Americas (*n* = 2), Africa (*n* = 1), Asia (*n* = 3), and Australia (*n* = 2).

Of the 11 genomes, two (FDA952758-C1-A and FDA952758-C1-C) were from strains isolated from blanched peanut kernels in 2016 in China, and both had the same ST3300 profile as strains from France. Of the remaining nine strains, four had profile ST2831, and the others had profiles ST5123 (*n* = 2), ST2333 (*n* = 1), ST2900 (*n* = 1), and ST579 (*n* = 1). The four ST2831 genomes were: isolated from spices in 2013 in India (*n* = 1), human strains from the United States (US) isolated in 2015 (*n* = 2), and a human strain from the United Kingdom (UK) isolated in 2019 (*n* = 1).

Hierarchical clustering (HC) cgMLST analysis performed by EnteroBase showed that all 53 *S*. Welikade strains belonged to the same super-lineage (HC2000_468). HierCC clustering is based on the genomic distances calculated using the number of shared cgMLST alleles, with single-linkage clustering criteria. Maximum allelic differences are indicated by the suffix of each HC, starting with HC0 for 0 cgMLST allelic differences, other than missing data, through to HC2850 for 2850 allelic differences [22].

According to SISTR serotype prediction [24], we found among the 53 HC2000_468 genomes, 52 assigned to *S*. Welikade and 1 to a monophasic variant of *S*. Welikade (16:l,v:-), as well as 400 to *S*. Gaminara. The 53 *S*. Welikade genomes and the first 10 *S*. Gaminara genomes, that had the lowest number of contigs, were selected for EnteroBase cgMLST analysis. The cgMLST tree was visualized by a minimum spanning tree produced using the EnteroBase GrapeTree [25] tool (Supplementary Table 1 and Supplementary Fig. 2). Among the ten *S*. Gaminara genomes, two were complete: SA20063285 [NCBI_NZ_CP030288.1] and CFSAN070644 [NCBI_ NZ_CP024165.1]. The panel of 53 *S*. Welikade strains harbored 2,414 and 3,904 alleles of difference with SA20063285 and CFSAN070644, respectively (Supplementary Fig. 2). Since the quality of these two Gaminara complete genomes are similar (> 50X coverage, high N50 and *Salmonella* correct genome size comprised between 4.5 and 5.5 Mb)) we decided to select the reference genome regarding to the cgMLST distances value. Finally, the complete *S*. Gaminara genome SA20063285 was chosen as the reference genome for the variant calling phylogenetic SNP core-genome analysis because it is the closest to Welikade genomes (Supplementary Table 1, Supplementary Fig. 2 and Supplementary Fig. 3).

**Fig. 1:**
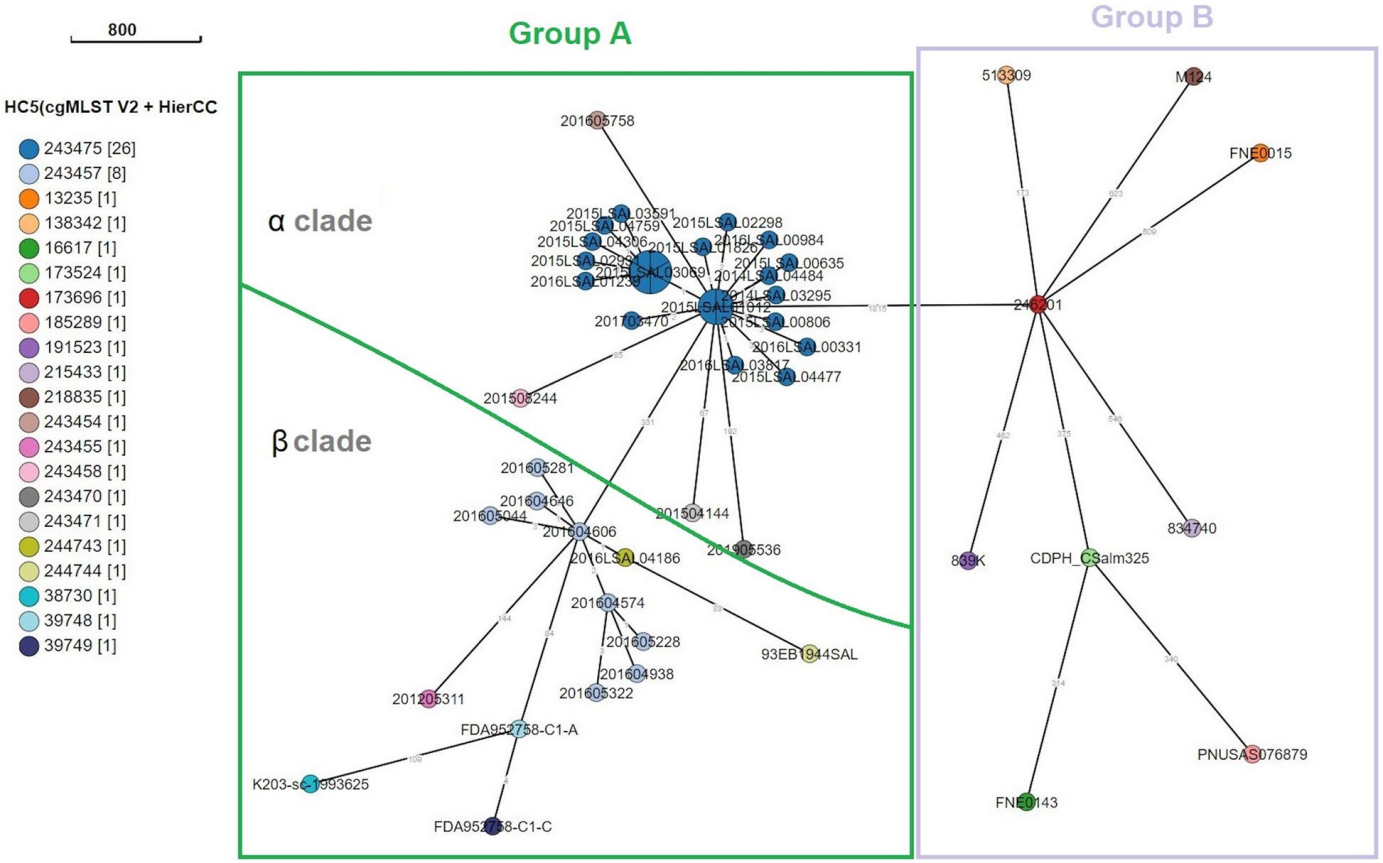
EnteroBase GrapeTree clustering of cgMLST allelic distances between S. Welikade strains. Clustering was produced using EnteroBase GrapeTree with the MSTreeV2 algorithm. Allelic distances are indicated on branches. Different colored nodes indicate the Hierarchical Clustering (HC)-5 profiles

EnteroBase cgMLST analysis was carried out on the 53 *S*. Welikade genomes alone. The spanning tree obtained revealed two different groups: group A, characterized by HC 900_38730 (ST3300 and ST2333) and including genomes from France, China and Nigeria; and group B, characterized by HC 900_13235 (ST579, ST2831, ST2900, ST5123 and ST6416) and including genomes from the UK, the US, India, Australia, and Sri Lanka (Supplementary Fig. 4). The HC5 analysis revealed two clades within group A (Fig. 1). The 25 strains isolated from the poultry sector in France (22 from *G. gallus* and 3 from feed) in Auvergne-Rhône-Alpes between 2015 and 2019 grouped together in a clade (α clade) with four human strains from France. The eight human strains related to the goat’s cheese outbreak in France in the summer of 2016 grouped with two strains isolated from goat’s cheese in the Occitanie region, one human strain isolated in France in 2012, two strains from China from blanched peanut kernels isolated in 2016, and one strain from Nigeria from a non-specified source (β clade).

### SNP calling analyses

#### EnteroBase

**Fig. 2:**
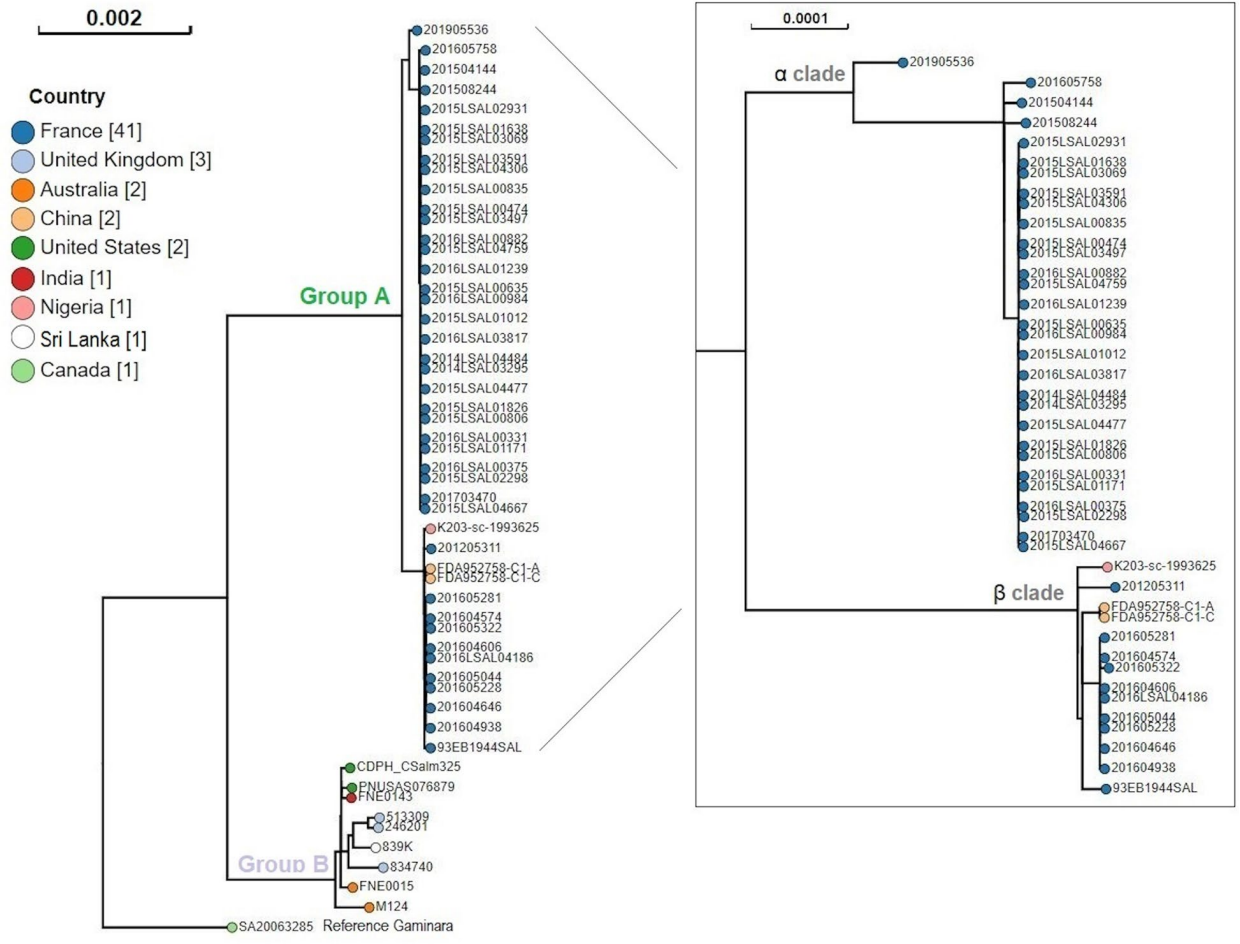
EnteroBase dendrogram of non-repetitive SNPs of the 53 S. Welikade genomes and the S. Gaminara reference genome SA20063285. The dendrogram was produced using EnteroBase SNP project tools. Different colored nodes indicate the strains’ geographic localization. The number of strains per country is indicated between brackets [n]

The EnteroBase SNP analysis performed on the 53 *S*. Welikade genomes, using *S.* Gaminara genome SA20063285 as a reference, revealed 42,521 variant sites. The two groups (groups A and B) identified within the SNP-based dendrogram (Fig. 2) were in accordance with the results observed by cgMLST analysis. Group A included genomes from strains isolated in France, China and Nigeria. Group B included genomes from strains isolated in the UK, the US, India, Australia and Sri Lanka. Two clades (α and β) were grouped within group A, as previously described (Fig. 2).

#### iVARCall2

**Fig. 3:**
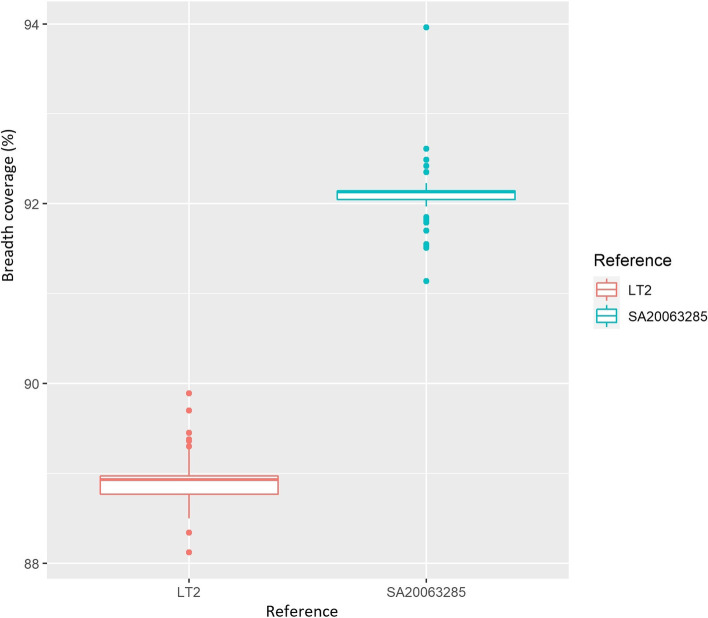
Boxplot representing breadth coverage data according to the reference genome used

SNP phylogenetic core-genome analysis was also carried out on the 52 *S*. Welikade genomes with the iVARCall2 pipeline, using *S*. Gaminara genome SA20063285 as a reference. The breadth coverage of the 52 Welikade genomes on the *S*. Gaminara SA20063285 genome used as a reference was 92.10%. As a comparison, a breadth coverage of 88.94% was calculated on the Typhimurium LT2 genome reference (Fig. 3).

The panel of 52 genomes consisted of the 42 genomes from France and 10 genomes available through the EnteroBase *Salmonella* database [14, 20]. Of the eleven *S*. Welikade genomes downloaded from EnteroBase, we excluded the FDA952758-C1-A genome because of its low sequence quality (genome fraction: 77%; assembly size: 3.7 Gb) (Supplementary Table 1).

To consider the effect of recombination on phylogenetic reconstruction, the ClonalFrameML tool was used to identify recombination events over the 52 *S*. Welikade genomes. ClonalFrameML analysis made it possible to identify 488 recombination events on all branches of the clonal genealogy (Supplementary Fig. 5). The length of recombined fragments ranged from 10 bp to 127,301 bp, and the mean length of imports was estimated to be δ = 2,444.23 bp. The average distance of the imports was ν = 0.00734. The ratio of rates of recombination and mutation was R/θ = 0.33, whereas the ratio of effects of recombination and mutation was r/m = 5.90. This indicated that recombination happened three times less often than mutation, although recombination overall caused six times more polymorphism than mutation. At the basal node that harbors group A, a hotspot of 202 recombination events was identified, with lengths ranging from 260 to 50,961 bp (dark blue horizontal line in Supplementary Fig. 5). A second hotspot of recombination events was identified at the basal node harboring group B, revealing 170 recombination events, with lengths ranging from 102 to 46,203 bp (dark blue horizontal line in Supplementary Fig. 5). Interestingly, one recombination event was identified in the α clade of group A. The length of this recombined fragment was 147 bp. No recombination events were observed in the β clade.

**Fig. 4:**
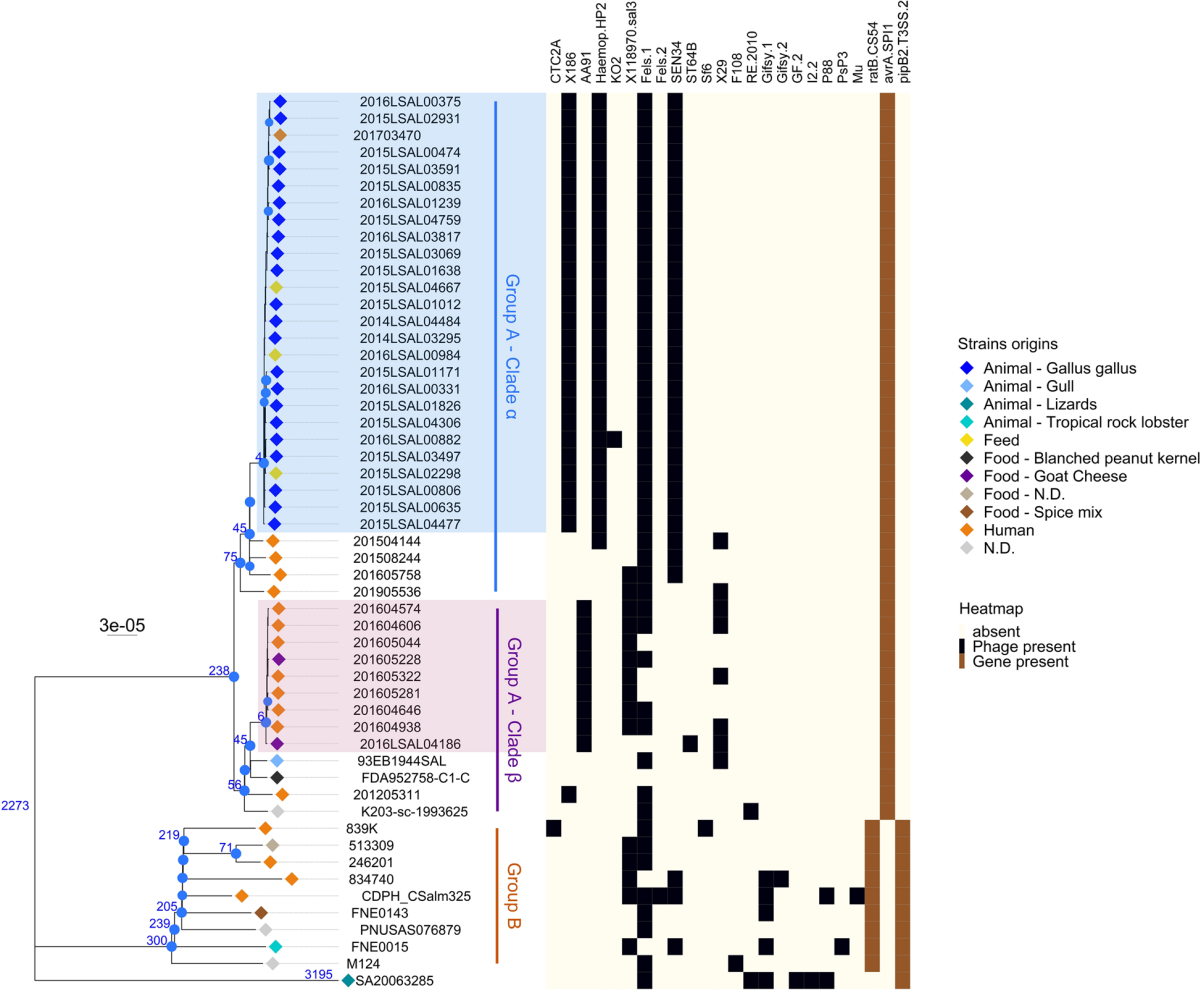
Phylogenetic tree based on the core-genome SNPs of S. Welikade strains, constructed with maximum likelihood according to the K3Pu + F + I model. Consensus tree was obtained after 3,000 bootstraps. SNP tree branch lengths were corrected taking in account the recombination events predicted by the ClonalFrame ML tool. The tree is rooted on the historical strain 839 K. The SNP average carried out on branches is noted in blue. Bootstrap values greater than 80% are noted with blue dots on nodes. The strains implicated in the goat’s cheese outbreak that occurred in France in 2016 are highlighted in the purple box. The blue box highlights the epidemic Gallus gallus cluster. The heatmap shows the presence (in black) or absence (in beige) of the phages. The accession numbers of each phage are: CTC2A [NC_ 030949], 186 [NC_001317], AA91 [NC_022750], Haemop_HP2 [ NC_003315], KO2 [NC_005857], 118970_sal3 [NC_031940], Fels-1 [NC_010391], Fels-2 [NC_010463], SEN34_[NC_028699], ST64B [NC_004313], Sf6 [NC_005344], X29 [NC_024369], F108 [NC_008193], RE-2010 _[NC_019488], Gifsy-1 _[NC_010392], Gifsy-2 _[NC_010393], GF-2 _[NC_026611], I2_2 _[NC_001332], P88 _[NC_026014], PsP3 _[NC_005340], and Mu _[NC_000929]

The iVarCall2 SNP core-genome phylogenetic analysis was carried out excluding recombination events. The tree obtained showed two groups (Fig. 4). The average distance within these two groups was 2,273 SNPs with a standard deviation (SD) of 61 SNPs. All strains in group A belonged to MLST profile ST3300, except for two strains from China and one from Nigeria, belonging to MLST profile ST2333.

Group A contained 43 genomes divided into two clades, both supported by a bootstrap value of 100%. These two clades (α and β) were genetically distant by an average of 238 SNPs ± 6 SD. The α clade contained 30 genomes, among which 26 were closely related with an average of 4 SNPs ± 2 SD. These 26 genomes corresponded to strains isolated between 2014 and 2017, with one human strain isolated in 2017 and 25 non-human strains isolated from the poultry sector: 22 strains isolated from *G. gallus* and 3 from feed (Fig. 4). The β clade contained 13 genomes harboring four different branches. One branch harbored 9 closely related strains with an average of 6 SNPs ± 4 SD. This cluster included seven human strains: five strains collected during the goat’s cheese outbreak of 2016 in south-western France (Occitanie region), and two other human strains isolated the same year from the south-west (Occitanie region) and south-east (Provence-Alpes-Côtes-d'Azur), respectively. These seven human strains were closely related to two strains isolated from goat’s cheese, with an average of 3 SNPs ± 1 SD and 12 SNPs ± 2 SD, respectively. The two strains isolated from goat’s cheese differed by 13 SNPs.

Group B contained the remaining 9 genomes, characterized by 5 different MLST profiles: ST579, ST2831, ST2900, ST5123, and ST6416. The average distance between these genomes was 235 SNPs ± 57 SD (Fig. 4).

**Fig. 5:**
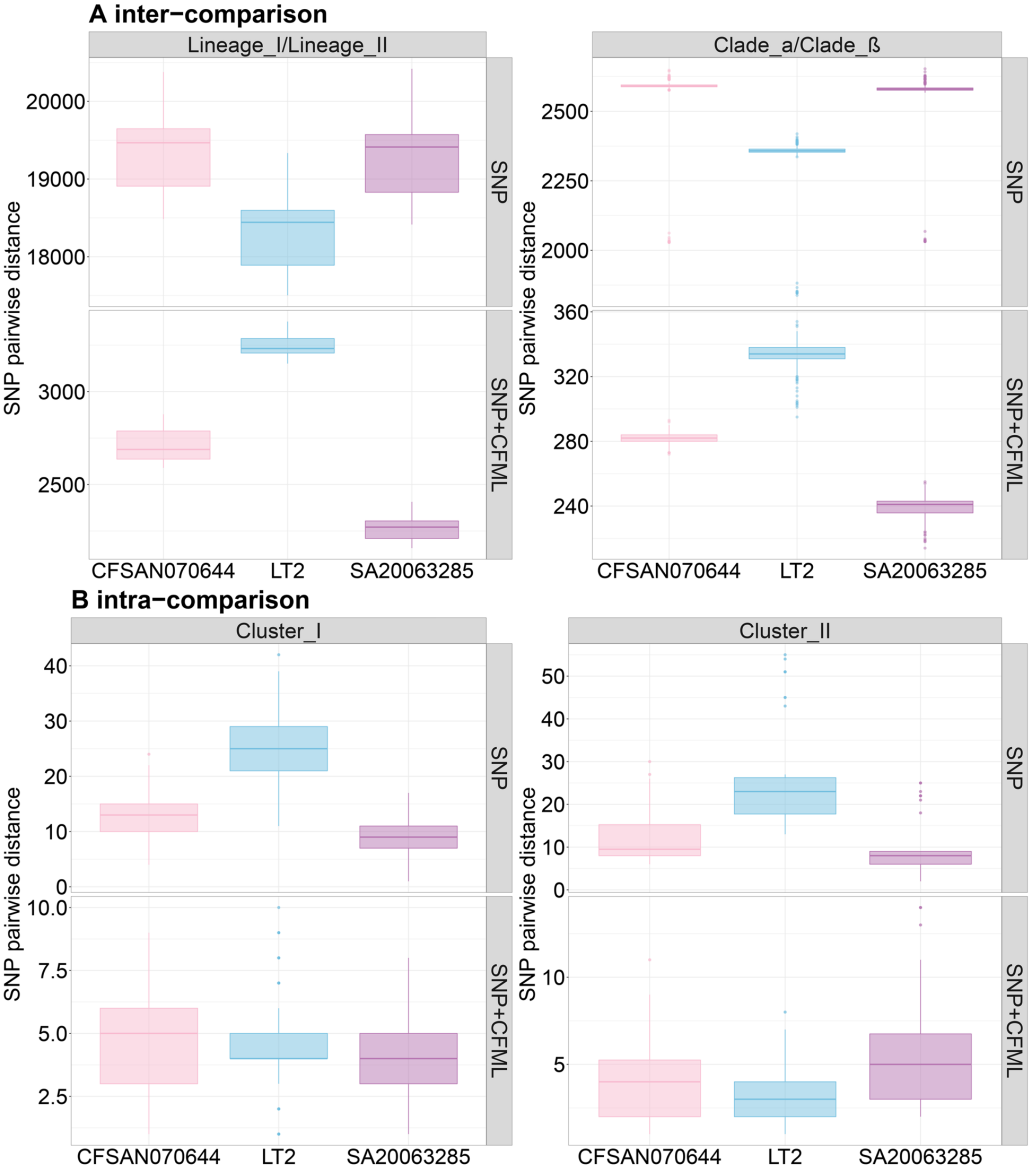
Boxplot representing the average SNP distance calculated as a function of the reference genome used and accounting or not for recombination events. **a** inter-comparison of SNP distance average between lineages or clades. **b** intra-comparison of SNP distance average inside clusters. Two conditions were compared: SNP and SNP + CFML. SNP: SNP analysis using the iVARCall2 tool; SNP + CFML: SNP analysis using iVARCall2 and a ClonalFrameML analysis. Three reference genomes were compared: Gaminara SA200663285, Gaminara CFSAN070644, and Typhimurium LT2

Comparison between phylogenetic SNP analyses with or without ClonalFrame using *S.* Gaminara SA20063285, *S*. Gaminara CFSAN070644 and LT2 Typhimurium genomes as references showed that extensive recombination events occurred within Welikade lineage LI/LII and clades α and β (Fig. 5a). This analysis also showed that to calculate SNP differences, the choice of reference genome was no longer critical if clustering analysis was carried out without recombination events. By contrast, with recombination events, the choice of the closest serovar to use for SNP phylogenetic analysis is critical. Although Gaminara serovar (e.i. the closest serovar to Welikade) provided the best clustering results. No critical differences were observed between the clustering results with two complete genomes *S.* Gaminara SA20063285 and *S*. Gaminara CFSAN070644 (Fig. 5b).

### Accessory-genome analysis

The results obtained with PHASTER software, a phage search tool, showed 21 intact prophages. All outbreak strains belonging to the β cluster (9 strains highlighted in purple in Fig. 4) carried Enterobacteria phage AA91-ss (NCBI NC 022,750) [26]. All strains isolated from the poultry sector belonging to the α cluster (22 strains highlighted in blue in Fig. 4) carried *Escherichia* phage 186 (NCBI NC 001,317) [27], *Haemophilus* phage HP2 (NCBI NC 003,315), and *Salmonella* phage SEN34 (NCBI NC 028,699). The *Salmonella* phage Fels-1 (NCBI NC 010,391) was identified within 46 *S*. Welikade genomes (Fig. 4).

The antibiotic resistance gene analysis, performed through the ResFinder database [28], showed that all Welikade genomes harbored the aac(6')-Iaa_1_NC gene (coding for aminoglycoside N-acetyltransferase). No other antibiotic resistance genes inventoried in the ResFinder database were detected in our panel of genomes.

The virulome analysis, performed through the vfdb database [29], showed the presence of 101 genes within the genomes of our dataset (Supplementary Table S2). The virulence genes found in *S*. Welikade strains are also mostly present in the *S*. Gaminara reference genome SA20063285 [NCBI_NZ_CP030288.1]. Only gene differences are highlighted in the heat map of Fig. 4. The *avrA* gene*,* which is part of the *Salmonella* Pathogenic Island-1 (SPI-1), was only found within group A. The *ratB* and *pipB2* genes were only found within group B; they are part of the CS54 island and SPI-2, respectively (Fig. 4).

The *Salmonella* pathogenicity island (SPI) analysis carried out on the 52 *S*. Welikade genomes compared to Typhimurium LT2 [NCBI_NZ_ AE006468.2] (i.e. one of the most common serovars in *Salmonella* outbreaks worldwide) showed the presence of several genes contained in SPI-1, SPI-2, SPI-3, SPI-4, SPI-5, SPI-6, SPI-12, SPI-13, SPI-14, SPI-18, and CS54 (Supplementary Table S3). As described above with the vfdb results, within the CS54 and SPI-2 pathogenicity islands, the *ratB* and *pipB2* genes were only found in group B. Similarly, within the SPI-1 pathogenicity island, the *avrA* gene was only found in group A.

## Discussion

The choice of the reference strain, as closely related as possible to strains under investigation, is an essential prerequisite before launching an SNP analysis based on comparison with a reference strain. It is also important to take a critical look at the quality of the reference genome used. Coverage is frequently considered as the main quality metric typically used in WGS and the value of 50 × has been chosen for *Salmonella* in the recommendations of the European Centre for Disease Control and Prevention (ECDC) [30]. Good coverage prevents poor MLST, cgMLST and, antigenic data and contributes to the correct clustering analysis for outbreaks and source attribution investigations [17, 30, 31]. Furthermore, availability of reads, information on genome length, serovar prediction and MLST type are also valuable information to consider when choosing reference genome [30]. When there is no complete genome available corresponding to the serovar of the investigated strains, it is essential to identify the closest serovar. Although this step is known to be crucial, it is rarely described in the literature [32]. The choice of a reference genome close to the strains under study increases the fraction of the genome on which the search for SNP variants will be carried out, thus increasing method sensitivity. Use of the reference genome Typhimurium LT2 would lead us to lose 11% of core genome information (89% of breadth coverage). The choice of *S.* Gaminara strain SA20063285 as the reference allowed us to lose only 8% of core genome information (92% breadth coverage). The choice of the reference genome is a critical step to ensure the sensitivity of the analyses performed when analyzing closely related genomes [33]. Nevertheless, for the Welikade genomes analyses in this study, recombination events heavily affected the clustering analysis and SNP differences calculation. Use of the ClonalFrame tool allowed us to bypass this problem.

EnteroBase Hierarchical clustering analysis at the HC2000 level (super-lineage) allowed us to easily determine the closest serovar to *S*. Welikade and thus to find the most suitable reference genome for SNP calling analysis. Moreover, submitting our genomes on EnteroBase enabled us to look at the population structure of *S*. Welikade, and to include 11 additional genomes from other countries in our analysis. Finally, the EnteroBase cgMLST and SNP analyses were found to be fast, user-friendly tools for outbreak clustering pre-investigations. Nevertheless, for dataset studied, SNP phylogenetic core-genome analysis carried out with an iVARCall2 workflow enabled finer clustering between strains and better calculation of genomic distances between genomes. SNP core-genome analysis revealed high genomic diversity among the *S.* Welikade strains analyzed. Two distinct groups were identified, genetically distant by an average of 2,273 SNPs. The *S*. Gaminara genome SA20063285, used as a reference, was genetically distant from the *S*. Welikade genomes in our dataset by an average of 3,195 SNPs.

Therefore, the *S*. Welikade phylogenetic core-genome analysis confirmed that the five strains isolated from patients in south-western France in 2016 were closely related to the two goat’s cheese strains, and revealed that two other human strains not recorded as part of the investigation (average of 16 SNPs, 4 SD) were also part of this outbreak. Interestingly, these other two human strains, 201,605,322 and 201,605,281, were also isolated in July 2016, the first from the Occitanie region (i.e. region where the outbreak was declared in summer 2016) and the second from the Provence-Alpes-Côtes-d'Azur, a region bordering the Occitanie. Moreover, all the strains isolated from the poultry sector (from animals and from feed) between 2014 and 2016 were genetically close (average of 4 SNPs, 2 SD). This result suggests a link between feed and poultry flocks, underlining the link between farm management and incidence of animal infection. Otherwise, one human case was related to them: human strain 201,703,470 isolated from a patient in 2017. Our genomic analysis made it possible to relate this human sporadic case to the poultry sector, even though further analyses would be needed to understand the links between this infection and the poultry strains.

The analysis to identify recombination events pointed out two major hotspots of recombination at the basal node of the two groups identified by the SNP analysis. These results suggest likely differentiation within the *S.* Welikade population into two distant genomic groups as the result of two major recombination events. Otherwise, the strains belonging to each cluster were clonal. These two genomic groups were also identified by the accessory genome analysis. The first genomic group, including strains from France, China and Nigeria, was characterized by the absence of genes *ratB* and *pipB2*. The second genomic group, including strains from the UK, US, India, Australia, and Sri Lanka, was characterized by the absence of the *arvA* gene. The *ratB* gene is described as part of CS54, a *Salmonella* genomic island. The outer membrane protein encoded by this gene seems to be involved in *S.* Typhimurium adherence and colonization of the cecum [34]. The absence of this gene has already been described in epidemic *S*. Derby ST40 strains in France [35]. The *pipB2* gene is part of the SPI-2 type III secretion system (SPI-2 T3SS) and likely plays a role as an effector in host cell vacuole colonization [36]. Finally, the *arvA* gene is part of SPI-1. This gene is described as lost in the arizonae subspecies [37]. Higher diversity was observed in phage profiles, which allowed us to identify two profiles within genomic group A, consistent with phylogenetic clustering. One profile including all strains from the poultry sector in France was characterized by phages X186, HP2, Fels-1, and SEN34; the second profile including the strains involved in the goat’s cheese outbreak that occurred in France in 2016 was characterized by phage AA91-ss and X118970. Interestingly, AA91-ss [38] is a bacteriophage already described in *Escherichia coli* O157:H7, carrying 3 *cdt* genes coding for a cytolethal toxin. It may be assumed that the *S*. Welikade genomes isolated from goat’s cheese have acquired this phage by transduction events, and this could lead to a selective advantage.

This is the first genomic study on *S.* Welikade. It allowed us to acquire new knowledge about this serovar, both in terms of epidemiology and genomics. The genomic position of *S*. Welikade within the *Salmonella* genomes available in EnteroBase was described. We were able to characterize the human and non-human *S*. Welikade strains in France. We were also able to relate the human infections that occurred during an outbreak in 2016 and sporadic cases that occurred over seven years to food sources.

The results obtained from accessory genome analysis confirmed that this approach is useful to distinguish genomes, particularly in the case of studies involving strains of unexpected origin, either strains of poorly described serovars with little pathogenicity for humans, or strains isolated from unusual hosts.

Genomes that are made available on public databases such as EnteroBase14 may therefore prove useful for future studies on this poorly referenced serovar.

## Conclusions

A comparison between genomes identified in France and those registered in EnteroBase was used to select the appropriate reference genome for a highly discriminative SNP phylogenetic analysis. The genomic analysis scheme proposed that calculating distances between the *S*. Gaminara and *S*. Welikade serovars may prove useful for further investigations. This is particularly the case for other serovars that are poorly referenced, and/or for which there is no complete genome available in the databases.

Our findings make it possible to propose a scheme to carry out highly discriminant SNP phylogenetic analyses for serovars, such as *S*. Welikade, for which no reference genome is available. The approach described here requires further study to build a sound foundation for genomic assessment of rare serovars. The ultimate aim is to better identify the sources of infections caused by these rare serovars, contributing to public health, and promoting a more preventive approach in frameworks of integrative surveillance.

## Methods

### Selection of isolates for retrospective epidemiological investigations

Forty *Salmonella enterica* subsp. *enterica* Welikade strains were selected for the retrospective genomic analysis of the outbreak. The data concerning these strains are presented in Supplementary Table 1**.** Among these genomes, 27 strains were selected from the ANSES Salmonella Network (SN) collection, comprising 23 strains isolated from animals (of which 22 strains from *Gallus gallus*) 1 strain isolated from food (strain isolated in south-eastern France in 2016 from goat’s cheese), and 3 strains isolated from feed for poultry.

Finally, 13 strains were selected from the French National Reference Center (NRC) for *Escherichia coli*, *Shigella* and *Salmonella* at the Institut Pasteur in Paris. One strain was isolated from goat’s cheese and 12 strains were isolated from humans: 5 strains from patients identified as part of the 2016 outbreak, 6 strains isolated between 2012 and 2016 in different regions of France, and the historical strain isolated in 1956 from a patient in Sri Lanka, which gave this serovar the name Welikade.

These 40 strains were identified as serovar Welikade by glass slide agglutination, according to the White–Kauffmann–Le Minor scheme [38].

### Genomic DNA preparation and sequencing

The 27 genomes from the ANSES collection and the 13 from Institut Pasteur were sequenced as previously described by Radomski et al. [39] and Ung et al. [40], respectively. The 40 strains were sequenced with Illumina technology.

### Genome quality control

The quality control, normalization, and assembly were performed with an in-house workflow called ARtWORK [41]. As previously described by Vila Nova et al., 2019 [42], the ARtWORK workflow is based on coverage control (i.e., > 100X) with Bbmap [43], read normalization (i.e., 100X) with Bbnorm [44], quality control of reads with FastQC [45], and read trimming (i.e., > 20 quality control) with Trimmomatic [46]. The quality rules followed were: (1) length of read must be higher than or equal to 50 base pairs (bp), otherwise excluded; (2) phred score per base must be higher than or equal to 30x; and (3) adapters must be filtered away based on an internal database with Illumina adapters. De novo assembly was performed through SPAdes [47]. Medusa and Gapcloser [48] were used to optimize and finish the assembly, as previously described by Palma et al., 2020 [49].

### Determination of the closest serovar

The 40 *S.* Welikade strains selected for the retrospective genomic analysis of the outbreak were uploaded into EnteroBase [22] with the corresponding metadata. The short reads were assembled by EnteroBase. Multilocus sequence typing (MLST), serovar prediction, and hierarchical clustering were performed automatically.

The EnteroBase *Salmonella* database [14, 20] was interrogated by searching the HC2000 profile of *S*. Welikade strains, following instructions described by Zhou et al. in 2020 [22]. GrapeTree clustering was used with the MSTreeV2 algorithm [25].

### Genome analysis for outbreak investigation

#### Multilocus sequence typing (MLST)

The seven housekeeping gene sequences (*aroC, dnaN, hemD, hisD, purE, sucA*, and *thrA*) for each strain were detected using EnteroBase [22] MLST tools, which enabled us to determine the sequence type (ST) directly from the fastq files.

#### SNP calling analysis

A dendrogram of non-repetitive SNP calling was produced within EnteroBase with the SNP project tool against the *S.* Gaminara genome SA20063285 [NCBI_NZ_CP030288.1] reference genome, with a cut-off set at a minimum sites present of 95%.

A core genome SNP analysis was conducted using the iVARCall2 workflow [50, 51]. Reads were mapped to both the *S.* Gaminara SA20063285 reference genome [NCBI_NZ_CP030288.1] and *S.* Typhimurium LT2 reference genome [NCBI_AE006468.2]. A pseudogenome obtained on the SA20063285 reference was generated using the HaplotypeCaller algorithm (GATK). SNPs were predicted, and the distance matrix between each pair of genomes was calculated.

#### Phylogenetic inference

The phylogenetic tree generated on the core genome dataset was computed using the IQ-TREE tool [52]. The tree was constructed based on the pseudogenome under the maximum likelihood criterion (ML). The model of nucleotide evolution was automatically selected by the ModelFinder [53] option according to the Bayesian Information Criterion (BIC). ModelFinder selected the three substitution-type models with unequal base frequency and invariable sites (K3Pu + F + I). The branch supports for the tree were estimated with 3,000 bootstrap replicates using the UFBoot [54] option (Ultrafast Boostrap Approximation).

#### Estimation of recombination

Recombination events were detected and the branch lengths were corrected taking into account the phylogenetic reconstruction obtained with the ClonalFrameML tool, as described by Diderot et al. in 2015 [55]. We used as input files the ML tree generated using the IQ-TREE tool and the pseudogenome file obtained using the iVARCall2 workflow. The R/theta rate, ratio of frequency of recombination and mutation, was directly obtained in the ClonalFrameML output. The r/m rate, ratio of effects of recombination and mutation, was calculated using the formula r/m = R/theta*delta*nu.

In order to produce a SNP distance matrix excluding variants linked to recombination events (> 400 bp), the script ‘Clonal_VCFilter’ [51] was applied.

The phylogenetic inference was corrected, accounting for the detected recombinations. Trees were visualized and annotated using R package ggtree [56, 57].

#### Phage identification

The presence of phage sequences in the assembly of *S.* Welikade strains was investigated using the PHASTER online application [58]. Only prophages identified as “intact” were considered. The identity of all intact prophage sequences detected by PHASTER was confirmed by BLAST [59]. A heatmap of the presence/absence of phages in the genomes studied was produced using R [56], with the ggtree package [57].

#### Identification of Salmonella pathogenicity island (SPI), virulence factor, and acquired resistance genes

The SPI analysis was carried out as described by Sévellec et al., 2018, with a cut-off set at a minimum coverage of 80% and minimum 90% identity. The presence/absence of genes mediating antibiotic resistance and virulence factors was investigated on the assembly using the GENIAL workflow [60]. This workflow made it possible to perform a blast via the ABRICATE application [61] against the ResFinder database [28] available at the Center for Genomic Epidemiology (CGE) (Denmark) and virulence factor database (vfdb) [29] available from the Institute of Pathogen Biology (Beijing, China). The ABRICATE outputs show only the genes found on at least one genome of the analyzed panel. The threshold for reporting a match between a gene in the databases and the input *Salmonella* genome was set at a minimum coverage of 80% and minimum 90% identity.

## Supplementary Information

**Additional file 1: Figure S1.** Sankey’s data frame and geographic distribution of the *S*. Welikade strains from France analyzed.

**Additional file 2: Figure S2.** EnteroBase GrapeTree of cgMLST allelic distance between genomic entries with HC2000 profile 468. Allelic distances are indicated on branches. Different colored nodes indicate the predicted serovars (SISTR1). The cgMLST tree includes 53 S. Welikade strains, including one monophasic S. Welikade strain (pink node), and ten S. Gaminara strains. S. Gaminara complete genomes CFSAN070644 and SA20063285 are indicated. The SA20063285 genome was chosen as a reference for the SNP phylogenetic analysis.

**Additional file 3: Figure S3.** Decision tree to select the reference genome for SNP analyses when complete genome is not available for serovar of interest. Enterobase *Salmonella* database (at https://enterobase.warwick.ac.uk/species/index/senterica) log in is required before starting. Light blue and purple blocks describe the actions to carry up to reference genome selection with specific instruction in italic indicated by a cog icon. At the right, red boxes display selection following SISTR and SeqSero2 results. The focus points are indicated by an eye icon in blue boxes.

**Additional file 4: Figure S4.** GrapeTree (Zhou et al. [25]) of cgMLST allelic distances between S. Welikade strains. Nodes are colored according to their geographic localization. Allelic distances are indicated on branches.

**Additional file 5: Figure S5.** Representation of recombination events for each branch and node of the phylogenetic inference of the S. Welikade genomes. Recombination corrected maximum likelihood tree are shown on the left. The yellow dotted lines indicate the nodes with a high number of recombination events. To the right of the tree, dark blue horizontal bars show recombination events along the concatenated genome segments. Invariant sites are shown in light blue (i.e., the background). White bars indicate non-homoplastic nucleotide substitutions. The increasing level of redness indicates the increasing degree of probable homoplastic nucleotide substitutions (Didelot & Wilson, [25]).

**Additional file 6: Table S1.** Epidemiological, assembly and genomics data for the *S*. Welikade panel.

**Additional file 7: Table S2.** Virulome study results obtained by vfdb. The table presents all the genes recorded in the vfdb database identified in the strains studied.

**Additional file 8: Table S3.** Virulome study results obtained from the private Bionumerics^®^ virulome database. Comparison of presence/absence of virulence genes among the three *S*. Welikade groups and in the *S.* Typhimurium LT2 genome. Differences are noted in bold.

## Data Availability

Genomics sequence used in this project are available online on the NCBI network under the accession: PRJNA734902 (available at: http://www.ncbi.nlm.nih.gov/bioproject/734902). Genomic assemblies used in this project are available online in the EnteroBase *Salmonella* database. Details for each genomic data are summarized in Supplementary Table 1, including the accession numbers for each strain.

## Abbreviations

cgMLST: Core genome MLST
DNA: Desoxyribonucleic acid
MLST: Multilocus sequence typing
NRC: National Reference Center
SD: Standard deviation
SN: *Salmonella* Network
SNP: Single -nucleotide polymorphism

## References

[1] European Food Safety Authority; European Centre for Disease Prevention and Control. The European Union summary report on trends and sources of zoonoses, zoonotic agents and food‐borne outbreaks in 2015. EFSA J. 2016;14(12):e04634. 10.2903/j.efsa.2017.5077 PMC7009962 32625371

[2] European Food Safety Authority; European Centre for Disease Prevention and Control. The European Union summary report on trends and sources of zoonoses, zoonotic agents and food-borne outbreaks in 2016. EFSA J. 2017;15(12):e05077. 10.2903/j.efsa.2017.5077 PMC7009962 32625371

[3] European Food Safety Authority; European Centre for Disease Prevention and Control. The European Union summary report on trends and sources of zoonoses, zoonotic agents and food-borne outbreaks in 2017. EFSA J. 2018;16(12):e05500. 10.2903/j.efsa.2018.5500 PMC7009540 32625785

[4] European Food Safety Authority; European Centre for Disease Prevention and Control. The European Union One Health 2018 Zoonoses Report. EFSA J. 2019;17(12):e05926. 10.2903/j.efsa.2019.5926 PMC7055727 32626211

[5] European Food Safety Authority; European Centre for Disease Prevention and Control. The European Union One Health 2019 Zoonoses Report. EFSA J. 2021;19(2):e06406. 10.2903/j.efsa.2021.6406 PMC7913300 33680134

[6] Centre National de Référence des Escherichia coli Shigella et Salmonella, Institut Pasteur; Laboratoire associé Service de Microbiologie, Hôpital Robert Debré, Paris, Rapport d’activité annuel - Année d’exercice 2016. 2016. Available online at https://www.pasteur.fr/sites/default/files/rubrique_pro_sante_publique/les_cnr/escherichia_coli_shigella_salmonella/rapport-cnr_escherichia-coli-shigella-salmonella-2016_pdf_final_.pdf

[7] Centre National de Référence des Escherichia coli Shigella et Salmonella, Institut Pasteur; Laboratoire associé Service de Microbiologie, Hôpital Robert Debré, Paris, Rapport d’activité annuel 2018 - Année d’exercice 2017. 2018. Available online at https://www.pasteur.fr/fr/file/21346/download.

[8] Centre National de Référence des Escherichia coli Shigella et Salmonella, Institut Pasteur; Laboratoire associé Service de Microbiologie, Hôpital Robert Debré, Paris, Rapport d’activité annuel 2019 - Année d’exercice 2018. 2019. Available online at https://www.pasteur.fr/fr/file/30716/download.

[9] Centre National de Référence des Escherichia coli Shigella et Salmonella, Institut pasteur; Laboratoire associé Service de Microbiologie Hôpital Robert Debré, Paris, Rapport d’activité annuel 2020 - Année d’exercice 2019. 2020. Available online at https://www.pasteur.fr/fr/file/40811/download.

[10] Velaudapillai T, Nitiananda K, Meedeniya K (1963). Salmonella in Desiccated Coconut. Z Hyg Infektionskr.

[11] Iveson JB, Bradshaw SD, How RA, Smith DW (2014). Human migration is important in the international spread of exotic Salmonella serovars in animal and human populations. Epidemiol Infect.

[12] Boqvist S, Hansson I, Nord Bjerselius U, Hamilton C, Wahlstrom H, Noll B, Tysen E, Engvall A (2003). Salmonella isolated from animals and feed production in Sweden between 1993 and 1997. Acta Vet Scand.

[13] Clark K, Karsch-Mizrachi I, Lipman DJ, Ostell J, Sayers EW (2016). GenBank. Nucleic Acids Res.

[14] Alikhan NF, Zhou Z, Sergeant MJ, Achtman M (2018). A genomic overview of the population structure of Salmonella. PLoS Genet.

[15] Lailler R, Moury F, Granier SA, Brisabois A (2012). The Salmonella Network, a tool for monitoring Salmonella “from farm to fork”. Euroreference.

[16] Salmonella Network; https://www.anses.fr/fr/content/inventaire-des-salmonella-dorigine-non-humaine.

[17] Nadon C, Van Walle I, Gerner-Smidt P, Campos J, Chinen I, Concepcion-Acevedo J, Gilpin B, Smith AM, Man Kam K, Perez E, Trees E, Kubota K, Takkinen J, Nielsen EM, Carleton H, Panel F-NE (2017). PulseNet International: Vision for the implementation of whole genome sequencing (WGS) for global food-borne disease surveillance. Euro Surveill.

[18] Mughini-Gras L, Kooh P, Fravalo P, Augustin JC, Guillier L, David J, Thebault A, Carlin F, Leclercq A, Jourdan-Da-Silva N, Pavio N, Villena I, Sanaa M, Watier L (2019). Critical Orientation in the Jungle of Currently Available Methods and Types of Data for Source Attribution of Foodborne Diseases. Front Microbiol.

[19] Sevellec Y, Granier SA, Le Hello S, Weill FX, Guillier L, Mistou MY, Cadel-Six S (2020). Source Attribution Study of Sporadic Salmonella Derby Cases in France. Front Microbiol.

[20] Achtman M, Zhou Z, Alikhan NF, Tyne W, Parkhill J, Cormican M, Chiou CS, Torpdahl M, Litrup E, Prendergast DM, Moore JE, Strain S, Kornschober C, Meinersmann R, Uesbeck A, Weill FX, Coffey A, Andrews-Polymenis H, Curtiss Rd R, Fanning S (2020). Genomic diversity of Salmonella enterica -The UoWUCC 10K genomes project. Wellcome Open Res.

[21] Gossner CM, Le Hello S, de Jong B, Rolfhamre P, Faensen D, Weill F-X, Giesecke J (2016). Around the World in 1,475 Salmonella Geo-serotypes. Emerg Infect Dis.

[22] Zhou Z, Alikhan NF, Mohamed K, Fan Y, Achtman M, Agama Study G (2020). The EnteroBase user's guide, with case studies on Salmonella transmissions, Yersinia pestis phylogeny, and Escherichia core genomic diversity. Genome Res.

[23] Achtman M, Wain J, Weill FX, Nair S, Zhou Z, Sangal V, Krauland MG, Hale JL, Harbottle H, Uesbeck A, Dougan G, Harrison LH, Brisse S, SEMS Group (2012). Multilocus sequence typing as a replacement for serotyping in Salmonella enterica. PLoS Pathog.

[24] Yoshida CE, Kruczkiewicz P, Laing CR, Lingohr EJ, Gannon VP, Nash JH, Taboada EN (2016). The Salmonella In Silico Typing Resource (SISTR): An Open Web-Accessible Tool for Rapidly Typing and Subtyping Draft Salmonella Genome Assemblies. PLoS One.

[25] Zhou Z, Alikhan NF, Sergeant MJ, Luhmann N, Vaz C, Francisco AP, Carrico JA, Achtman M (2018). GrapeTree: visualization of core genomic relationships among 100,000 bacterial pathogens. Genome Res.

[26] Allue-Guardia A, Imamovic L, Muniesa M (2013). Evolution of a self-inducible cytolethal distending toxin type V-encoding bacteriophage from Escherichia coli O157:H7 to Shigella sonnei. J Virol.

[27] Portelli R, Dodd IB, Xue Q, Egan JB (1998). The late-expressed region of the temperate coliphage 186 genome. Virology.

[28] Zankari E, Hasman H, Cosentino S, Vestergaard M, Rasmussen S, Lund O, Aarestrup FM, Larsen MV (2012). Identification of acquired antimicrobial resistance genes. J Antimicrob Chemother.

[29] Chen L, Zheng D, Liu B, Yang J, Jin Q (2016). VFDB 2016: hierarchical and refined dataset for big data analysis–10 years on. Nucleic Acids Res.

[30] European Centre for Disease Prevention and Control, Expert Opinion on the introduction of next-generation typing methods for food- and waterborne diseases in the EU and EEA. Stockholm: ECDC. 2015. Available online at https://www.ecdc.europa.eu/sites/default/files/media/en/publications/Publications/food-and-waterborne-diseases-next-generation-typing-methods.pdf.

[31] Zhang S, den Bakker HC, Li S, Chen J, Dinsmore BA, Lane C, Lauer AC, Fields PI, Deng X, Dudley EG (2019). SeqSero2: Rapid and Improved Salmonella Serotype Determination Using Whole-Genome Sequencing Data. Appl Environ Microbiol.

[32] Pightling AW, Petronella N, Pagotto F (2014). Choice of reference sequence and assembler for alignment of Listeria monocytogenes short-read sequence data greatly influences rates of error in SNP analyses. PLoS One.

[33] Bush SJ, Foster D, Eyre DW, Clark EL, De Maio N, Shaw L, Stoesser N, Peto TEA, Crook DW, Walker AS (2020). Genomic diversity affects the accuracy of bacterial single-nucleotide polymorphism-calling pipelines. Gigascience.

[34] Kingsley RA, Humphries AD, Weening EH, De Zoete MR, Winter S, Papaconstantinopoulou A, Dougan G, Baumler AJ (2003). Molecular and phenotypic analysis of the CS54 island of Salmonella enterica serotype typhimurium: identification of intestinal colonization and persistence determinants. Infect Immun.

[35] Sevellec Y, Vignaud ML, Granier SA, Lailler R, Feurer C, Le Hello S, Mistou MY, Cadel-Six S (2018). Polyphyletic Nature of Salmonella enterica Serotype Derby and Lineage-Specific Host-Association Revealed by Genome-Wide Analysis. Front Microbiol.

[36] Jennings E, Thurston TLM, Holden DW (2017). Salmonella SPI-2 Type III Secretion System Effectors: Molecular Mechanisms And Physiological Consequences. Cell Host Microbe.

[37] Shariat NW, Timme RE, Walters AT (2021). Phylogeny of Salmonella enterica subspecies arizonae by whole-genome sequencing reveals high incidence of polyphyly and low phase 1 H antigen variability. Microb Genom.

[38] Grimont PAD, Weill F-X. Antigenic Formulae of the Salmonella serovars. 9th Edition. World Health Organization Collaborating Center for Reference and Research on Salmonella, Institut Pasteur, Paris. 2007. Available online at: https://www.pasteur.fr/sites/default/files/vf_0.pdf.

[39] Radomski N, Cadel-Six S, Cherchame E, Felten A, Barbet P, Palma F, Mallet L, Le Hello S, Weill FX, Guillier L, Mistou MY (2019). A Simple and Robust Statistical Method to Define Genetic Relatedness of Samples Related to Outbreaks at the Genomic Scale - Application to Retrospective Salmonella Foodborne Outbreak Investigations. Front Microbiol.

[40] Ung A, Baidjoe AY, Van Cauteren D, Fawal N, Fabre L, Guerrisi C, Danis K, Morand A, Donguy MP, Lucas E, Rossignol L, Lefevre S, Vignaud ML, Cadel-Six S, Lailler R, Jourdan-Da Silva N, Le Hello S (2019). Disentangling a complex nationwide Salmonella Dublin outbreak associated with raw-milk cheese consumption, France, 2015 to 2016. Euro Surveill.

[41] Felten, A.; Durimel, K., ARtWORK. https://github.com/afelten-Anses/ARtWORK.

[42] Vila Nova M, Durimel K, La K, Felten A, Bessieres P, Mistou MY, Mariadassou M, Radomski N (2019). Genetic and metabolic signatures of Salmonella enterica subsp. enterica associated with animal sources at the pangenomic scale. BMC Genomics.

[43] Bushnell B, Rood J, Singer E (2017). BBMerge - Accurate paired shotgun read merging via overlap. PLoS One.

[44] Xu S, Ackerman MS, Long H, Bright L, Spitze K, Ramsdell JS, Thomas WK, Lynch M (2015). A Male-Specific Genetic Map of the Microcrustacean Daphnia pulex Based on Single-Sperm Whole-Genome Sequencing. Genetics.

[45] Andrews, S., FastQC: a quality control tool for high throughput sequence data. Babraham Bioinformatics. https://www.bioinformatics.babraham.ac.uk/projects/fastqc/ 2014.

[46] Bolger AM, Lohse M, Usadel B (2014). Trimmomatic: a flexible trimmer for Illumina sequence data. Bioinformatics.

[47] Bankevich A, Nurk S, Antipov D, Gurevich AA, Dvorkin M, Kulikov AS, Lesin VM, Nikolenko SI, Pham S, Prjibelski AD, Pyshkin AV, Sirotkin AV, Vyahhi N, Tesler G, Alekseyev MA, Pevzner PA (2012). SPAdes: a new genome assembly algorithm and its applications to single-cell sequencing. J Comput Biol.

[48] Kremer FS, McBride AJA, Pinto LS (2017). Approaches for in silico finishing of microbial genome sequences. Genet Mol Biol.

[49] Palma F, Brauge T, Radomski N, Mallet L, Felten A, Mistou MY, Brisabois A, Guillier L, Midelet-Bourdin G (2020). Dynamics of mobile genetic elements of Listeria monocytogenes persisting in ready-to-eat seafood processing plants in France. BMC Genomics.

[50] Felten A, Vila Nova M, Durimel K, Guillier L, Mistou MY, Radomski N (2017). First gene-ontology enrichment analysis based on bacterial coregenome variants: insights into adaptations of Salmonella serovars to mammalian- and avian-hosts. BMC Microbiol.

[51] Felten, A.; Durimel, K., Scripts for SNPs/INDELs analysis. https://github.com/afelten-Anses/VARtools.

[52] Nguyen LT, Schmidt HA, von Haeseler A, Minh BQ (2015). IQ-TREE: a fast and effective stochastic algorithm for estimating maximum-likelihood phylogenies. Mol Biol Evol.

[53] Kalyaanamoorthy S, Minh BQ, Wong TKF, von Haeseler A, Jermiin LS (2017). ModelFinder: fast model selection for accurate phylogenetic estimates. Nat Methods.

[54] Hoang DT, Chernomor O, von Haeseler A, Minh BQ, Vinh LS (2018). UFBoot2: Improving the Ultrafast Bootstrap Approximation. Mol Biol Evol.

[55] Didelot X, Wilson DJ (2015). ClonalFrameML: efficient inference of recombination in whole bacterial genomes. PLoS Comput Biol.

[56] Gentleman RC, Carey VJ, Bates DM, Bolstad B, Dettling M, Dudoit S, Ellis B, Gautier L, Ge Y, Gentry J, Hornik K, Hothorn T, Huber W, Iacus S, Irizarry R, Leisch F, Li C, Maechler M, Rossini AJ, Sawitzki G, Smith C, Smyth G, Tierney L, Yang JY, Zhang J (2004). Bioconductor: open software development for computational biology and bioinformatics. Genome Biol.

[57] Yu G, Smith DK, Zhu H, Guan Y, Lam TTY, McInerny G (2016). ggtree: anrpackage for visualization and annotation of phylogenetic trees with their covariates and other associated data. Methods Ecol Evol.

[58] Arndt D, Grant JR, Marcu A, Sajed T, Pon A, Liang Y, Wishart DS (2016). PHASTER: a better, faster version of the PHAST phage search tool. Nucleic Acids Res.

[59] Camacho C, Coulouris G, Avagyan V, Ma N, Papadopoulos J, Bealer K, Madden TL (2009). BLAST+: architecture and applications. BMC Bioinformatics.

[60] Barbet, P., GENIAL. https://github.com/p-barbet/GENIAL.

[61] Seemann, T., Abricate. https://github.com/tseemann/abricate.

