# Supplementary figures and images for: Salmonella enterica subsp. enterica Welikade: guideline for phylogenetic analysis of serovars rarely involved in foodborne outbreaks
Source: BMC Genomics. 2022 Mar 19;23:217. doi: 10.1186/s12864-022-08439-2 (PMC8933937; doi:10.1186/s12864-022-08439-2)

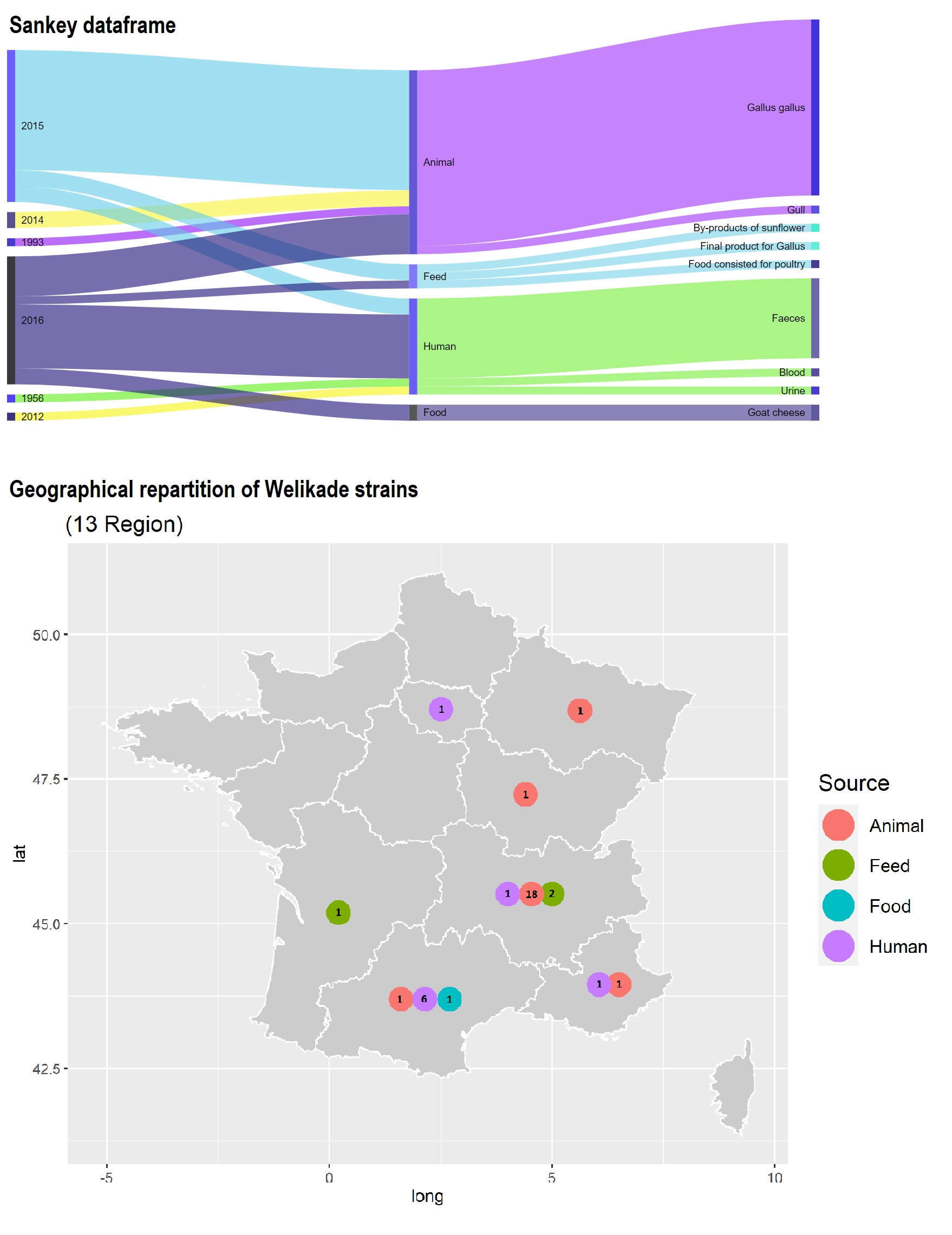

Supplement: Supplementary file 1 — Additional file 1: Figure S1. Sankey’s data frame and geographic distribution of the S. Welikade strains from France analyzed. [file 12864_2022_8439_MOESM1_ESM.jpg]

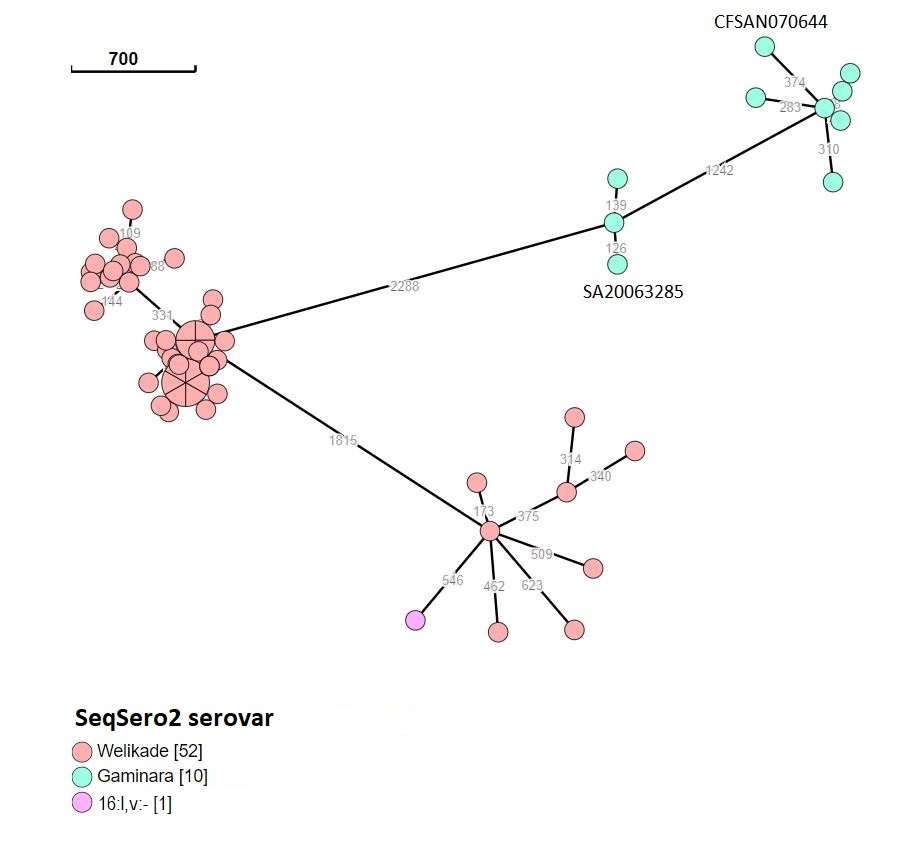

Supplement: Supplementary file 2 — Additional file 2: Figure S2. EnteroBase GrapeTree of cgMLST allelic distance between genomic entries with HC2000 profile 468. Allelic distances are indicated on branches. Different colored nodes indicate the predicted serovars (SISTR1). The cgMLST tree includes 53 S. Welikade strains, including one monophasic S. Welikade strain (pink node), and ten S. Gaminara strains. S. Gaminara complete genomes CFSAN070644 and SA20063285 are indicated. The SA20063285 genome was chosen as a reference for the SNP phylogenetic analysis. [file 12864_2022_8439_MOESM2_ESM.jpg]

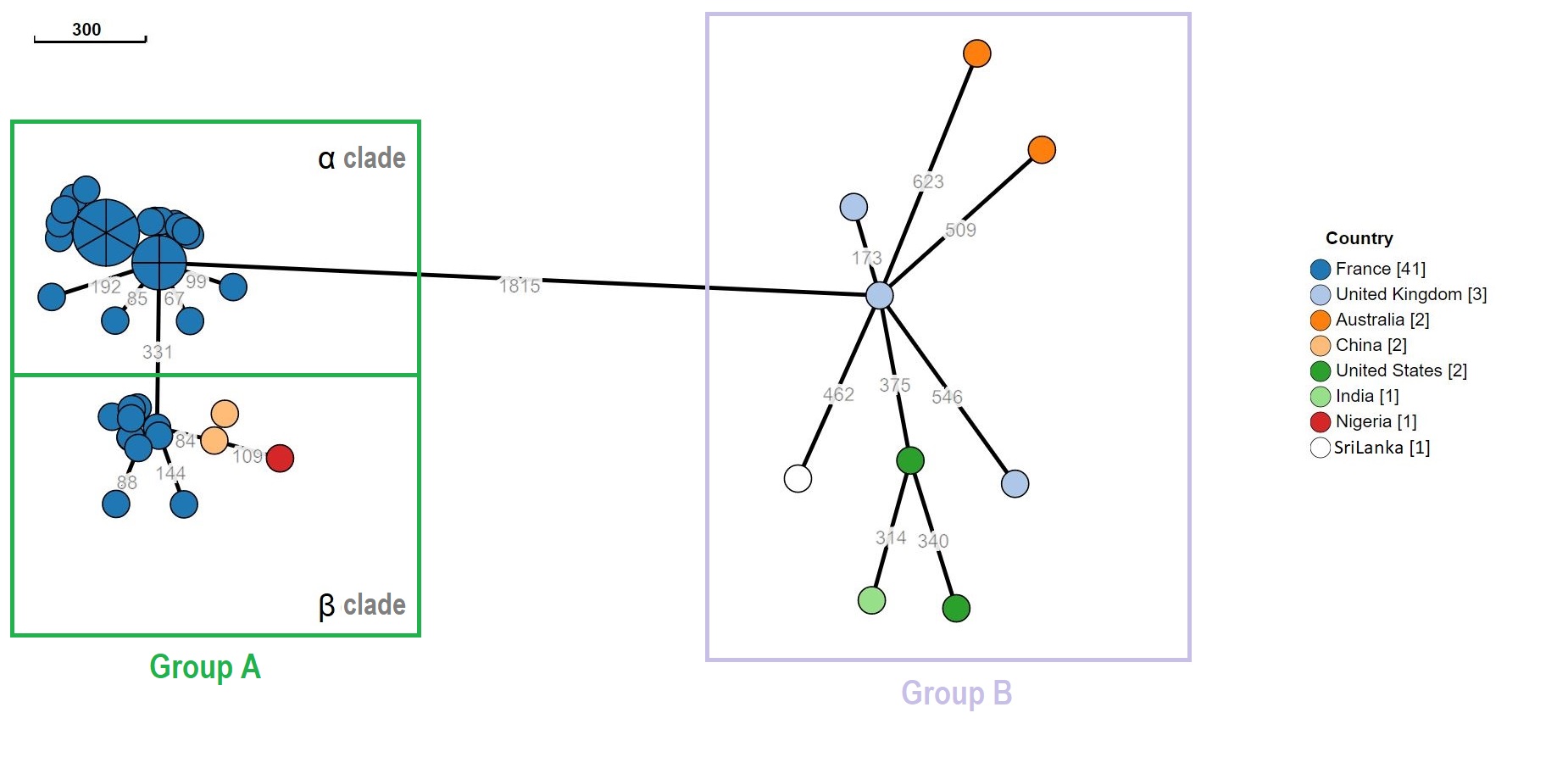

Supplement: Supplementary file 4 — Additional file 4: Figure S4. GrapeTree (Zhou et al. [25]) of cgMLST allelic distances between S. Welikade strains. Nodes are colored according to their geographic localization. Allelic distances are indicated on branches. [file 12864_2022_8439_MOESM4_ESM.jpg]

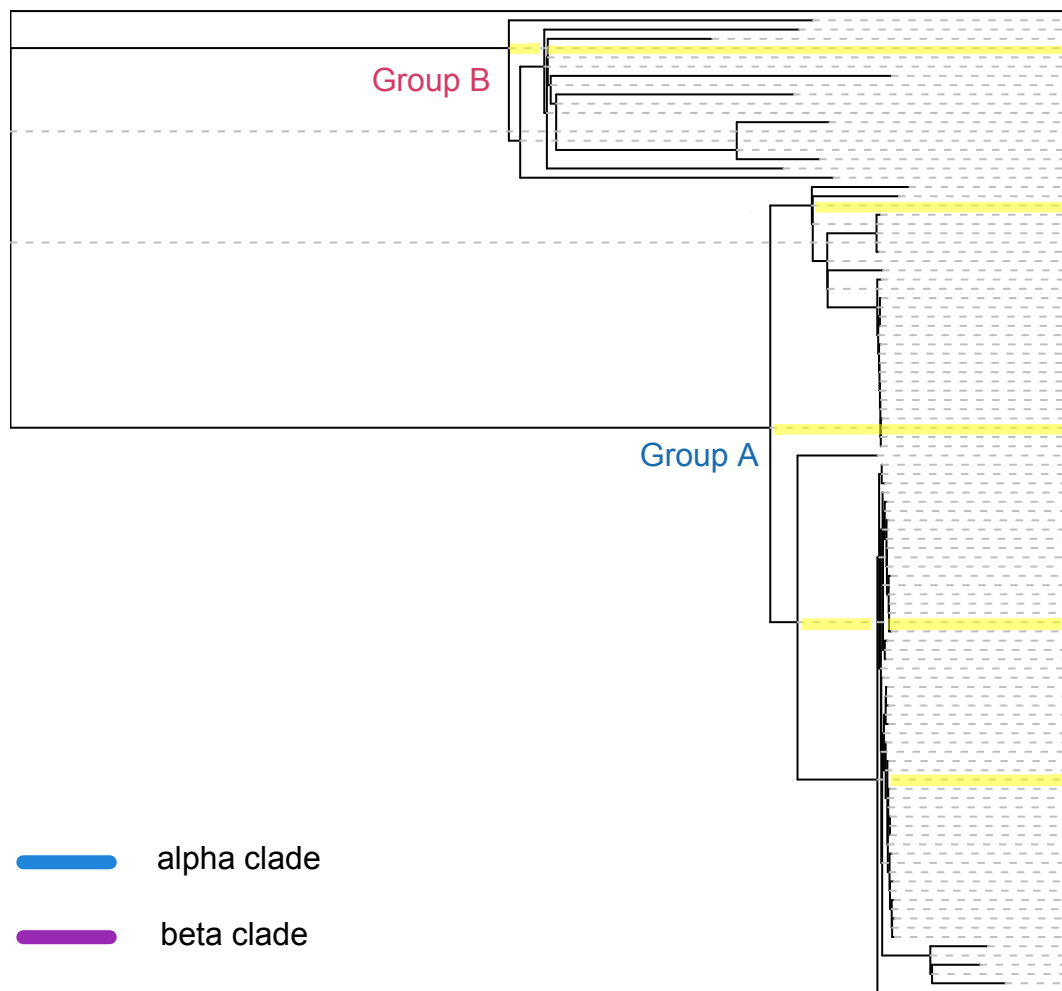

alpha clade

beta clade

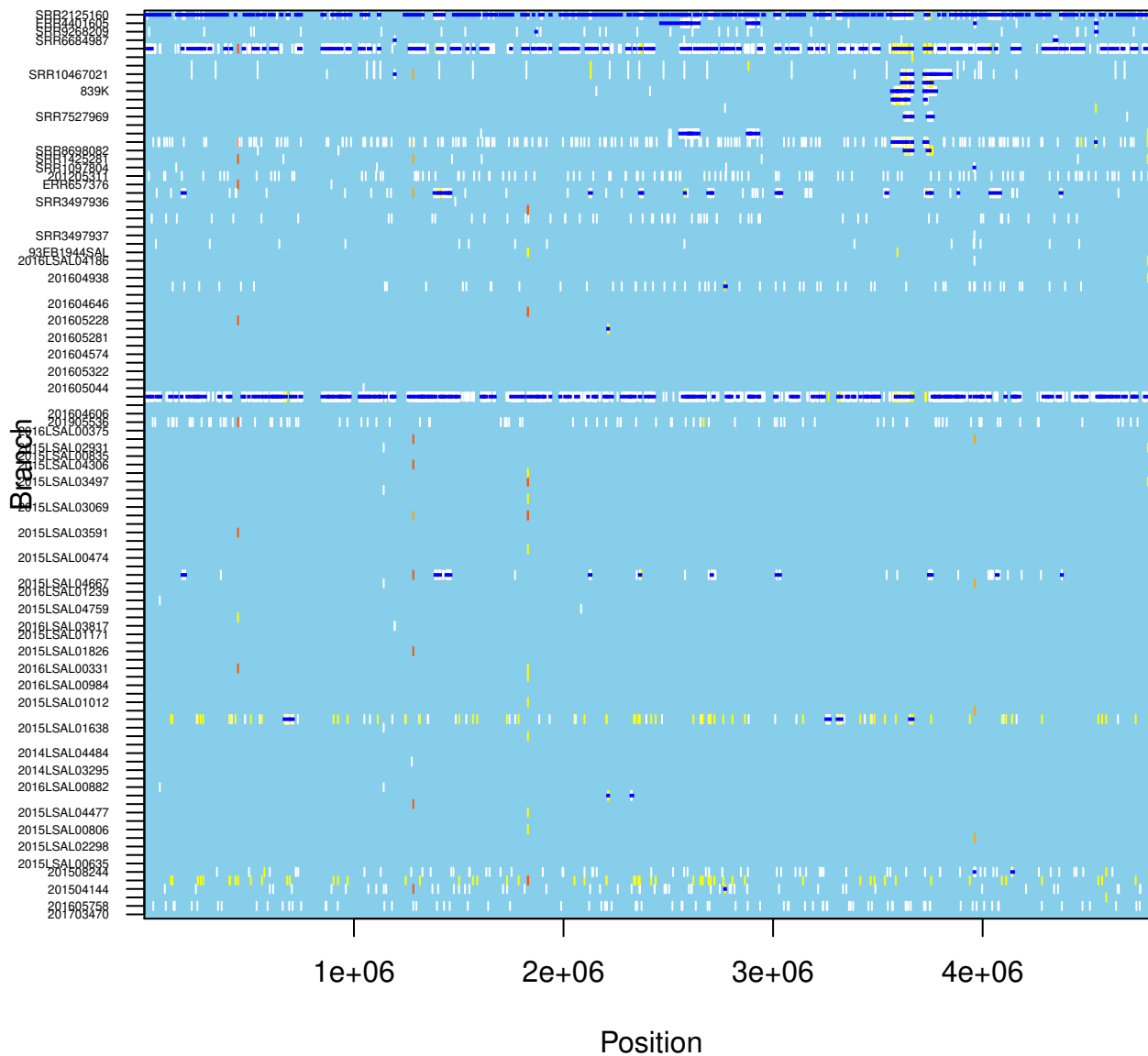

Supplement: Supplementary file 5 — Additional file 5: Figure S5. Representation of recombination events for each branch and node of the phylogenetic inference of the S. Welikade genomes. Recombination corrected maximum likelihood tree are shown on the left. The yellow dotted lines indicate the nodes with a high number of recombination events. To the right of the tree, dark blue horizontal bars show recombination events along the concatenated genome segments. Invariant sites are shown in light blue (i.e., the background). White bars indicate non-homoplastic nucleotide substitutions. The increasing level of redness indicates the increasing degree of probable homoplastic nucleotide substitutions (Didelot & Wilson, [25]). [file 12864_2022_8439_MOESM5_ESM.pdf]
