# Supplementary material for: Salmonella enterica subsp. enterica Welikade: guideline for phylogenetic analysis of serovars rarely involved in foodborne outbreaks
Source: BMC Genomics. 2022 Mar 19;23:217. doi: 10.1186/s12864-022-08439-2 (PMC8933937; doi:10.1186/s12864-022-08439-2)
Supplement: Supplementary file 3 — Additional file 3: Figure S3. Decision tree to select the reference genome for SNP analyses when complete genome is not available for serovar of interest. Enterobase Salmonella database (at https://enterobase.warwick.ac.uk/species/index/senterica) log in is required before starting. Light blue and purple blocks describe the actions to carry up to reference genome selection with specific instruction in italic indicated by a cog icon. At the right, red boxes display selection following SISTR and SeqSero2 results. The focus points are indicated by an eye icon in blue boxes. [file 12864_2022_8439_MOESM3_ESM.pdf]

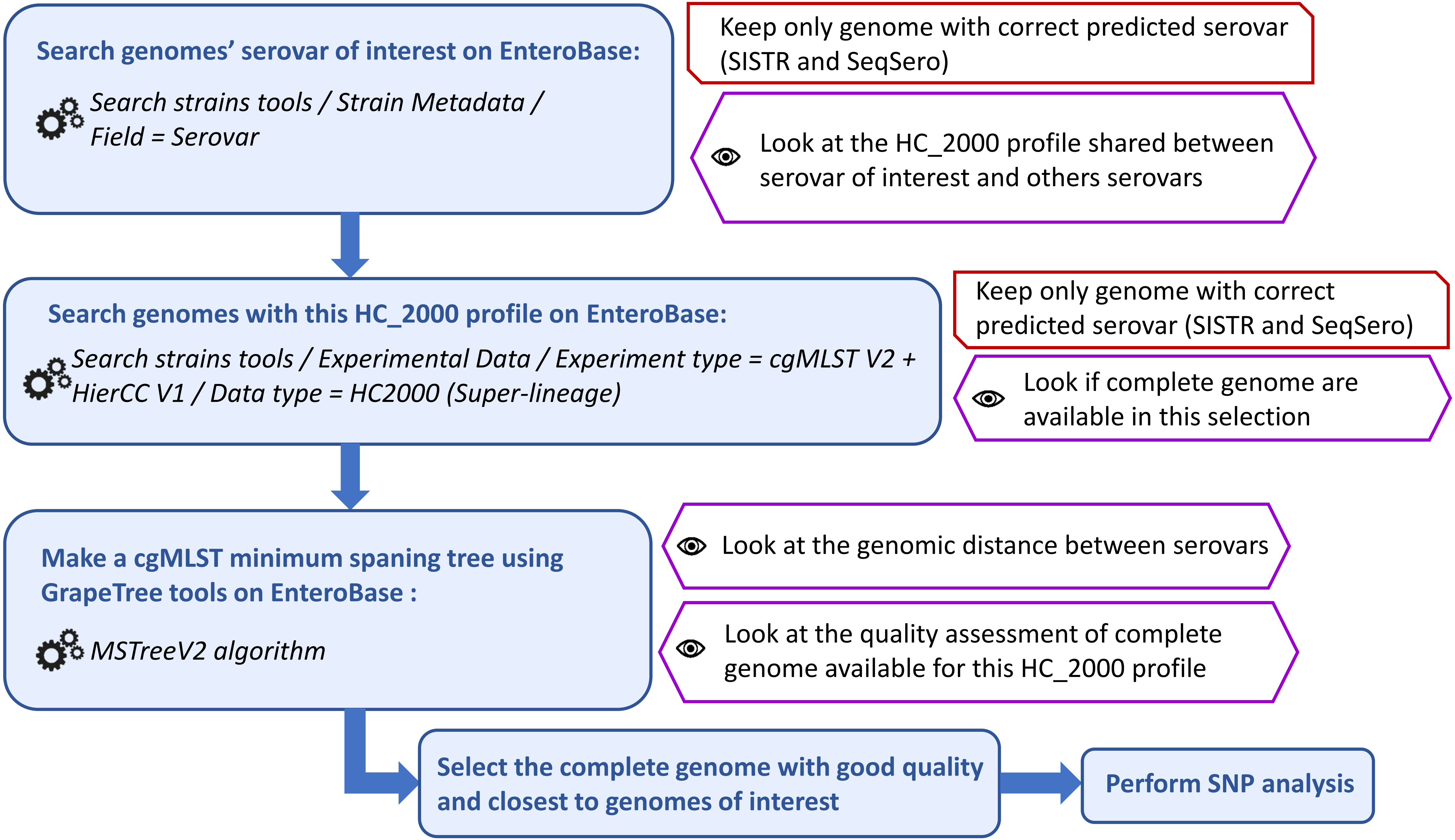
Search genomes' serovar of interest on EnteroBase:
Search strains tools / Strain Metadata / Field = Serovar
Keep only genome with correct predicted serovar (SISTR and SeqSero)
Look at the HC_2000 profile shared between serovar of interest and others serovars
Search genomes with this HC_2000 profile on EnteroBase:
Search strains tools / Experimental Data / Experiment type = cgMLST V2 + HierCC V1 / Data type = HC2000 (Super-lineage)
Keep only genome with correct predicted serovar (SISTR and SeqSero)
Look if complete genome are available in this selection
Make a cgMLST minimum spaning tree using GrapeTree tools on EnteroBase :
MSTreeV2 algorithm
Look at the genomic distance between serovars
Look at the quality assessment of complete genome available for this HC_2000 profile
Select the complete genome with good quality and closest to genomes of interest
Perform SNP analysis
